# Prevalence, severity and predictors of Antipsychotic-Related Adverse Drug Reactions at the Psychiatry Clinic of a Regional Referral Hospital in Uganda

**DOI:** 10.1371/journal.pone.0356000

**Published:** 2026-08-13

**Authors:** John Martin Tumwebaza, Sarad Pawar Naik Bukke, Bayapa Reddy Narapureddy, Radiana Makuza Kabera, Joel Sebisaalu, Amina Abubakar, Patrick Muasya Kitheka, Godwin Nimusiima, Awad Osman Abdalla Mohamed, Moses Muwanguzi, Scholastic Ashaba, Tadele Mekuriya Yadesa

**Affiliations:** 1 Department of Pharmacy, Faculty of Health Sciences, Victoria University, Kampala Uganda; 2 Department of Pharmaceutics and Pharmaceutical Technology, School of Pharmacy, Kampala International University, Ishaka, Uganda; 3 Department of Public Health, College of Applied Medical Sciences, King Khalid University, Abha, KSA; 4 Department of Anaesthesia and Operations, College of Applied Medical Sciences, King Khalid University, Abha, Saudi Arabia; 5 Department of Psychiatry, Faculty of Medicine, Mbarara University of Science and Technology, Mbarara, Uganda; 6 Department of Clinical Pharmacy and Pharmacy Practice, School of Pharmacy, Kampala International University, Ishaka, Uganda; Ekiti State University College of Medicine, NIGERIA

## Abstract

**Background:**

Adverse drug reactions (ADRs) are associated with hospitalization, increased healthcare cost, morbidity and mortality. This study aimed to investigate the prevalence, severity and associated factors of ADRs among patients taking antipsychotics at Mbarara Regional Referral Hospital (MRRH).

**Method:**

A cross-sectional study was conducted among patients taking antipsychotics at the psychiatric clinic from March to May 2025. Consecutive sampling was employed whereby all eligible patients attending the psychiatric clinic during the study period were recruited until the required sample size was attained. All patients aged 18 years and above, diagnosed with a psychotic disorder, and who had received an antipsychotic for at least the previous one month were interviewed. STATA version 17 was used for statistical data analysis. Descriptive statistics were presented as mean and standard deviation or median and interquartile ranges. ADRs were analyzed for severity using the modified Hartwig and Siegel scale. We run multivariable logistic regression analysis to determine factors associated with ADRs.

**Results:**

A total of 415 participants were included in the study with a mean age of 38. Fifteen ADRs were commonly reported among patients taking antipsychotics. More than half of the participants (256/415, 61.70%) experienced at least one ADR, with sedation being the most frequent (31.3%). Of the reported ADRs, 12/15 (80%) were moderate in severity. Separation from spouses (AOR = 2.01, 95% CI [1.08–3.77], *p* = 0.029), diagnosed with bipolar disorder (AOR = 2.09, 95% CI [1.07–4.07], *p* = 0.031), and co-administering both IM & oral antipsychotic (AOR = 1.95, 95% CI [1.03–3.70], *p* = 0.041) were significantly associated with ADRs.

**Conclusion:**

The current study showed that over 6 in 10 participants taking antipsychotics experienced at least one ADR, the majority of which were moderate in severity. Separation from a spouse, bipolar disorder, concomitant use of oral and intramascular antipsychotics were independently associated with ADRs. These findings highlight the need for strengthen monitoring and preventive strategies, particularly among high-risk patients taking antipsychotics. Integration of clinical pharmacists into the psychiatry care teams may further enhance ADR detection, monitoring and management.

## Introduction

Psychosis is a clinical syndrome characterized by hallucinations, delusions, grossly disorganized behavior and disorganized speech [1]. Globally, the prevalence of psychosis has been increasing by over 65% from 1990–2019 [2]. In sub-Saharan Africa, a scooping review revealed that the lifetime prevalence of psychosis varies from 1.0% to 4.4% [3]. In Uganda, a retrospective study carried out in Butabika National Referral Hospital (BNRH) in 2018, showed a prevalence of psychosis at 62.7% [4]. Patients with psychosis require antipsychotic medications to manage their symptoms and restore their functionality.

Antipsychotic drugs form a group of treatments primarily aimed at managing mental health conditions such as schizophrenia, anxiety and other disorders presenting with psychotic symptoms [5–7]. Antipsychotics are classified into first generation antipsychotics (FGAs), second generation antipsychotics (SGAs) [8] and third generation antipsychotics (TGAs) [8,9]. FGA for example haloperidol, trifluoperazine and chlorpromazine block dopamine (D2) receptors of dopaminergic neurons. SGAs primarily block serotonin receptors (5-HT2A) and D2 receptors and they include clozapine, quetiapine, olanzapine, and risperidone [8]. TGAs act by mainly partially agonizing D2 and 5HT1A receptors with examples including aripiprazole, cariprazine and brexpiprazole [10]. Adverse drug reactions have been observed to be common among most patients with psychosis taking antipsychotic medication.

The World Health Organization (WHO) defines an adverse drug reaction (ADR) as any harmful, unintended response to a medicine that occurs at doses normally used in man for the prevention, diagnosis or treatment of disease, or for modifying physiological function [11]. Globally, the prevalence of ADRs among patients taking antipsychotics has not been estimated in meta-analysis, however individual studies conducted in India and France have revealed an ADR prevalence of 5.67% and 8.3% respectively [12,13]. In sub-Saharan Africa, a meta-analysis revealed that the prevalence of ADRs among patients taking anti psychotics is at 51% [14]. In Uganda, and at Mbarara in particular, a study conducted among hospitalized patients revealed the prevalence of ADR at 48.9% [15]. No study, however, has been conducted to investigate ADRs among patients taking antipsychotics at psychiatric unit of MRRH. Examples of ADRs include sedation, weight gain, sexual dysfunction, tardive dyskinesia, akathisia, muscle stiffness, hyperglycemia, vomiting, dry mouth and slurred speech.

Factors that contribute to the development of ADRs can be drug related such as type of antipsychotic, other non-antipsychotic, polypharmacy, frequency, route of administration [16,17]. Patient related factors such as age, gender, level of education, marital status, occupation, income status and perceived social support may also lead to development of ADRs [18,19]. The other factors that play a role in the occurrence of ADRs are Illness related factors which include type of psychiatric disorder, duration with disorder, number of comorbid conditions, type of comorbid condition and number of in-hospital admission [20]. Age and polypharmacy are the most common predictors in development of ADR among patients taking antipsychotics [19,21,22].

ADRs contribute significantly to increased hospitalization [23] and medical costs [24]. A reported study in England showed that ADRs have an estimated admission cost of £490 716 which when extrapolated nationally, totals up to 2.21 billion annually [25]. ADRs also lead to therapy non-adherence and/or discontinuation [26] morbidity and mortality [27,28]. In USA, estimates indicate that ADRs rank as the fourth major contributor to mortality [29].

Despite the recognized global burden of antipsychotic-related ADRs, there remains a critical gag in Uganda, particularly in southwestern Uganda where Mbarara Regional Referral Hospital (MRRH) serves a population of over four million people. While previous studies at MRRH have examined ADRs among hospitalized patients with general medical conditions [15,30], none have specifically focused on antipsychotic-related ADRs among psychiatric outpatients. This gap is concerning given that MRRH’s psychiatric clinic serves approximately 100 patients weekly and over 1,200 new patients annually, with over 90% of outpatients prescribe antipsychotics.

Furthermore, the patient population at MRRH presents unique risk factors that have not been systematically studied. These include high utilization of first-generation antipsychotics (FGAs) due to government supply and affordability constraints, high prevalence of substance use disorders requiring higher antipsychotic doses and limited pharmacovigilance infrastructure.

This study, therefore, was designed to fill this evidence gap by instigating the prevalence, severity, and associated factors of ADRs among patients taking antipsychotics at the psychiatry clinic of MRRH. The findings will provide the foundational evidence needed to inform the development of targeted monitoring protocols, guide clinical decision-making regarding antipsychotic selections and dosing, support the integration of clinical pharmacists into psychiatry teams, and strengthen pharmacovigilance activities at the facility level.

## Methods

### Study design and setting

This was a cross-sectional study conducted among outpatients taking antipsychotics at Mbarara Regional Referral Hospital Psychiatric Unit between March 2025 to May 2025. MRRH, a 600-bed tertiary hospital, serves as a key referral hospital in southwestern Uganda, 270 kilometers from Kampala. It caters to a population exceeding four (4) million residents across districts including Mbarara, Bushenyi, Ntungamo, Kiruhura, Ibanda, Buhweju, Rubirizi, Mitooma, and Isingiro. Furthermore, it also provides care to patients from Kabale, Masaka, Fort Portal, and even from neighboring countries like Rwanda. The MRRH Psychiatric Clinic operates within the psychiatric ward on Tuesdays and Wednesdays. Normally, 100 patients are registered per week and approximately 1200 new patients annually. The clinic boasts a comprehensive team of mental health professionals, including psychiatrists (3), occupational therapists (3), counselor (1), social workers (2), psychiatric clinical officers (3), psychiatric nurses (3) and psychiatry residents (15).

### Eligibility criteria

#### Inclusion criteria.

We enrolled all patients at psychiatry outpatient clinic of MRRH, aged 18 years and above, who were diagnosed with a psychotic disorder, and currently taking antipsychotics for at least the past 1 month and who were willing to provide written informed consent for participation in the study.

#### Exclusion criteria.

We excluded all patients with active psychotic symptoms.

### Sample size determination

The sample size was calculated using Kish-Leslie formular; n = Z^2^P(1-P)/d^2^ [31]. The prevalence of ADR among patients taking antipsychotics in a previous study in India was 43.5% [32]. Since the study settings were similar, we used 43.5% as the expected prevalence (p) of ADR among patients taking antipsychotics, with a 0.05 significance (alpha) level at a 95% confidence interval (CI). *p* = 43.5% d = 0.05 z = 1.96 (at CI of 95%). Applying the formula mentioned above, the number of participants included in the study was = 378. A 10% non-response rate was added: = (10%*378) = 38. The target sample size that was interviewed = (378 + 38) = 415 participants.

A review of the number of patients attending the psychiatry clinic was conducted and on average 400 psychiatry patients are seen at the clinic per month.

### Sampling technique

A Consecutive sampling method was used until the sample size was attained, throughout the investigation. The procedure of gathering data took three months to complete to obtain the necessary sample size.

### Study variables

#### Dependent variable.

Adverse drug reactions were the primary outcome of this study. They included weight gain, akathisia, dry mouth, tremors, sedation, tardive dyskinesia, hyperglycemia, nausea and vomiting, increased appetite, slurred speech, constipation, skin rush, blurred vision, drowsiness, and dystonia. For the assessment of weight gain and hyperglycemia, baseline measurements were established using documented medical records or, where unavailable, structured patient/caregiver interviews. Baseline weight was defined as the patient’s body weight documented within the first week of initiating the current antipsychotic regimen or the earliest recorded weight prior to antipsychotic initiation. Weight gain was classified as an increase of 7% or more from this baseline weight, consistent with established clinical thresholds for antipsychotic-induced weight gain. For hyperglycemia, baseline glycemic status was determined by reviewing medical records for prior diagnoses of diabetes mellitus or documented elevated blood glucose prior to antipsychotic initiation, supplemented by patient and caregiver interviews to confirm the absence of pre-existing hyperglycemia. Newly elevated RBS was defined as a random blood sugar >180 mg/dL, and newly elevated FBS as a fasting blood sugar >100 mg/dL, occurring after antipsychotic initiation without prior documented hyperglycemia.

#### Operational definitions of ADRs assessed.

All 15 ADR types were assessed using standardized operational definitions derived from established pharmacovigilance guidelines and clinical assessment scales. Sedation was defined as subjective excessive sleepiness or reduced alertness interfering with daily functioning. Drowsiness was defined as abnormal sleepiness during waking hours. Tremors were defined as involuntary rhythmic oscillatory movements assessed through neurological examination. Weight gain was defined as an increase of ≥7% from baseline body weight established within the first week of antipsychotic initiation. Dry mouth was defined as subjective oral dryness attributed to antipsychotic therapy. Akathisia was defined as subjective inner restlessness with observable motor phenomena (fidgeting, pacing, inability to sit/stand still). Tardive dyskinesia was defined as new repetitive involuntary movements primarily affecting the oro-bucco-lingual area, developing after ≥3 months of antipsychotic exposure. Hyperglycemia was defined as new-onset elevation in blood glucose (RBS > 180 mg/dL or FBS > 100 mg/dL) occurring after antipsychotic initiation without prior documented diabetes mellitus. Nausea and vomiting were defined as subjective urge to vomit with or without expulsion of gastric contents. Increased appetite was defined as perceived increase in hunger beyond usual patterns. Slurred speech was defined as impaired articulation observed during clinical interviews. Constipation was defined as reduced bowel frequency (<3 per week) with difficulty passing stools. Skin rash was defined as new-onset cutaneous eruption excluding pre-existing dermatological conditions. Blurred vision was defined as subjective reduction in visual clarity. Dystonia was defined as sustained or intermittent involuntary muscle contractions causing abnormal postures or movements, assessed through clinical observation. Each ADR was confirmed by both the principal investigator and a resident psychiatrist before classification.

#### Independent variables.

The independent variables were factors that have been reported in the literature to be associated with ADRs among patients taking antipsychotics. These included socio-demographic characteristics, illness related factors and medication related factors [17,19–21,33].

Socio-demographic factors included age, gender, level of education, employment status, income status, marital status, perceived social support. Illness related factors included type of psychiatric disorder, duration with the disorder, number of comorbid conditions, type of the comorbid condition, number of in-patient hospital admission. The medication related factors included type of antipsychotic, route of administration, counselling on ADRs.

### Data collection tool, procedure, management, and quality control

The data collection instrument was a structured, interviewer-administered questionnaire comprising six distinct modules: (1) Socio-demographic characteristics (age, gender, education, marital status, employment, income), (2) Illness-related factors (type of psychiatric disorder, duration, comorbidities, hospital admissions), (3) Medication-related factors (antipsychotic type, route, duration, counselling, concurrent medications), (4) ADR profile assessment (presence of 15 specific ADRs with clinical criteria, onset timing, severity, causality, and preventability using Naranjo, Hartwig-Siegel, and Schumock-Thornton scales), and (6) Perceived social support (Multidimensional Scale of Perceived Social Support – MSPSS). Additionally, clinical assessments including weight measurement and blood glucose monitoring were performed where feasible. Perceived social support of the participants was assessed utilizing the Multidimensional Scale of Perceived Social Support (MSPSS) [34]. ADR was defined according to WHO’s definition of ADR as presented above [11]. The established adverse reaction of each drug was assessed using Up-To-Date (2024) version 3.66.4. ADRs were initially suspected when there was a relationship between drug administration and the onset and progression of the adverse reaction, after excluding other possible causes. Naranjo adverse drug reaction probability scale, a standard causality assessment tool, was used to determine the probability of ADR [35]. Severity of ADR was assessed using modified Hartwig and Siegel severity assessment Scale [36]. Preventability of ADR was evaluated using Schumock and Thornton preventability scale [37]. The Naranjo Probability Scale, Modified Hartwig and Siegel Severity Scale, and Schumock and Thornton Preventability Scale are well-established frameworks with high validity and reliability in pharmacovigilance [38].

Data collection followed a structured workflow with clearly defined personnel roles. The research team comprised the Principal Investigator (PI – a Clinical Pharmacist Resident with Master of Pharmacy in Clinical Pharmacy training), a Research Assistant (Psychiatry Resident), and a Psychiatric Nurse. All team members received standardized training on ethical procedures, data collection techniques, and ADR assessment using validated scales.

The data collection process involved: (1) screening and recruitment of eligible participants on clinic days (Tuesdays and Wednesdays); (2) obtaining written informed consent; (3) face-to-face interviews using a structured questionnaire with six modules (socio-demographics, illness factors, medication factors, ADR profiles, and social support); (4) medical record review for clinical and medication history; (5) clinical assessments including weight measurement, blood glucose monitoring (where feasible), and neurological examination for movement disorders.

For ADR assessment, suspected ADRs were initially identified by the PI based on pharmacological knowledge and patient history. Each suspected ADR was then jointly confirmed by the PI and the Resident Psychiatrist, considering temporal relationships, clinical presentation, and exclusion of other causes. Once confirmed, the Naranjo Adverse Drug Reaction Probability Scale was applied jointly to assess causality, the Modified Hartwig and Siegel Scale to classify severity, and the Schumock and Thornton Scale to determine preventability.

All completed questionnaires were reviewed daily by the PI for completeness and accuracy. Data were double-entered into a password-protected database and verified by the PI. A biostatistician provided support for data analysis using STATA Version 17.“

The research team included a clinical pharmacist, psychiatry residents and psychiatric nurses. The clinical pharmacist and psychiatry resident collected the data and assessed potential ADRs by considering the pharmacological effects of the patient’s prescribed drugs. The research team received instruction on ethical issues and data gathering procedures before the study began. Both English and Runyankole questionnaires were developed and were pretested among 10 patients at the psychiatry clinic at MRRH before the actual data collection begun. The principal investigator participated in data collection and verified data completeness daily throughout the entire data gathering period.

### Ethical considerations

#### Ethical approval and consent to participate.

The study was carried out in compliance with the Declaration of Helsinki. The study protocol was submitted for permission to conduct the study from the Department of Pharmacy, Faculty of Medicine Research Committee, Mbarara University Research Ethics Committee (MUST-REC) with a Reference No: MUST-2024–1960, and administrative approval was obtained from the director of Mbarara Regional Referral Hospital. In this study, participants were consecutively selected to voluntarily participate without any coercion after seeking informed consent. The participants also had an option to withdraw at any time without being castigated.

### Data analysis

STATA Version 17 was used to analyze the data. Data was presented in descriptive statistics for example for continuous variables that are normally distributed, mean ± Standard Deviation were used and for non-normally distributed continuous variables the median, inter-quartile range were used. Categorical variables were portrayed as frequencies with their corresponding percentages. Descriptive analysis was done for socio-demographics, drug-related and medication-related variables.

Objective 1: The prevalence of ADR among patients taking antipsychotics was determined by; dividing the number of patients experiencing ADRs (both at enrollment and during the study) by the total number of patients studied and this was presented as a percentage (%).

Objective 2: Severity of ADR was analyzed by using descriptive analysis and presented as a percentage of each category.

Objective 3: Bivariate and multivariate logistic regression analysis was applied to identify the independent factors associated with ADRs among the patients on antipsychotics. Variables that were biologically plausible were incorporated into the multivariate analysis if their bivariate analysis *p*-value was less than 0.25. Variables which had a *p*-value less than 0.05 were statistically significant.

## Results

### Socio-demographic characteristics of participants

A total of 415 participants were enrolled in this study. Their ages ranged from 18 to 90 years with the majority (303, 73.01%) aged between 26 and 60-years. More than half of the respondents were female (231, 55.7%). Most of participants were unemployed (77.1%), approximately one-third (34.2%) had attained secondary level education, and about two in five (41.9%) were married. Additionally, 328 participants (79.04%) reported high perceived social support (Table 1).

**Table 1:** Socio-demographic characteristics of participants (N = 415).

Variable	Category	Frequency	Percentage
**Age (years)** **Mean±SD (38 ± 14)**	18-25	81	19.52
	26-60	303	73.01
	>60	31	7.47
**Gender**	Female	231	55.7
	Male	184	44.3
**Level of education**	No formal education	30	7.2
	Primary	130	31.3
	Secondary	142	34.2
	Tertiary	113	27.2
**Marital status**	Single	164	39.5
	Married/Cohabiting	174	41.9
	Separated	77	18.6
**Employment status**	Formally employed	34	8.19
	Self-employed	60	14.46
	Unemployed	321	77.35
Estimated monthly income (in UGX)*	<300000	47	50.00
	300000-900000	29	30.85
	>900000	18	19.15
**Perceived social support**	Low	5	1.20
	Medium	82	19.76
	High	328	79.04

* are the participants who are formally and self-employed (n = 94).

### Clinical and medication use characteristics

Among the selected participants receiving antipsychotic therapy, the majority were diagnosed with bipolar disorder (187, 45.1%) with a median (IQR) illness duration of 2 (1,5) years. Only 2 (0.5%) of the participants had two comorbidities. HIV/AIDS was the most common (24, 5.8%) comorbid condition, followed by hypertension (17, 4.1%). All participants were on antipsychotic treatment, with most taking oral formulations (343, 82.7%), while 12 (2.9%) were receiving long-acting intramuscular (IM) antipsychotics, and 60 (14.5%) were on both oral and IM preparations (Table 2).

**Table 2:** Clinical characteristics of patients taking antipsychotics at MRRH.

Variable	Category	Frequency	Percentage
**Type of mental disorder**	Bipolar	187	45.1
	Depression	48	11.6
	Schizophrenia	126	30.4
	Substance use disorder	54	13.0
**Number of in-hospital admissions due to ADRs**	No	410	98
	One admission	3	0.72
	Two admissions	2	0.48
**Comorbidities**	None	370	89.2
	One	43	10.4
	Two	2	0.5
**HIV/AIDS**	No	391	94.2
	Yes	24	5.8
**Hypertension**	No	398	95.9
	Yes	17	4.1
**Diabetes mellitus**	No	409	98.6
	Yes	6	1.4
**Route of administration of antipsychotics**	Orally	343	82.7
	Both oral and IM	60	14.5
	IM	12	2.9
Type of oral antipsychotic*	First generation	322	79.9
	Second generation	81	20.1
**Counseled on ADRs**	No	48	11.57
	Yes	367	88.43

* are the participants who took oral and both oral with monthly IM injection (n = 403).

IM-Intramuscular, ADRs-Adverse drug reactions, HIV/AIDS-Human Immunodeficiency Virus/Acquired Immunodeficiency Syndrome.

Chlorpromazine (140, 43.5%) and risperidone (44, 54.3%) were the most commonly prescribed first and second-generation oral antipsychotics, respectively (Table 3). The median (IQR) duration on antipsychotics use was 20 (6, 48) months.

**Table 3:** Types of antipsychotics used by patients with mental illness at MRRH.

Variable	Category	Frequency	Percentage
**Oral first generation antipsychotics (n = 322)**	Chlorpromazine	140	43.5
	Haloperidol	128	39.8
	Trifluoperazine	53	16.5
	Thioridazine	1	0.3
**Parenteral long-acting first-generation antipsychotics (n = 71)**	Fluphenazine	43	60.6
	Haloperidol	15	21.1
	Flupentixol	8	11.3
	Zuclopentixol	5	7.0
**Oral second generation antipsychotics (n = 81)**	Risperidone	44	54.3
	Olanzapine	19	23.5
	Quetiapine	15	18.5
	Clozapine	3	3.7

### Prevalence of ADRs among patients taking antipsychotics

**Fig 1:**
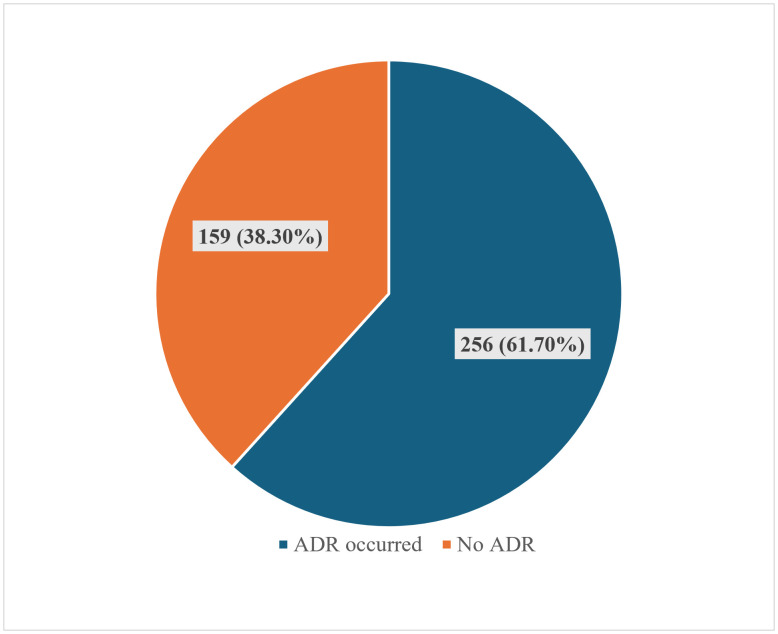
Prevalence of ADRs among patients taking antipsychotics at MRRH.

Out of the 415 participants, 256 experienced at least one ADR resulting in a prevalence of 61.70% (95% CI: 56.81% − 66.39%) (Fig 1). Among these, 110 participants reported two or more ADRs.

**Fig 2:**
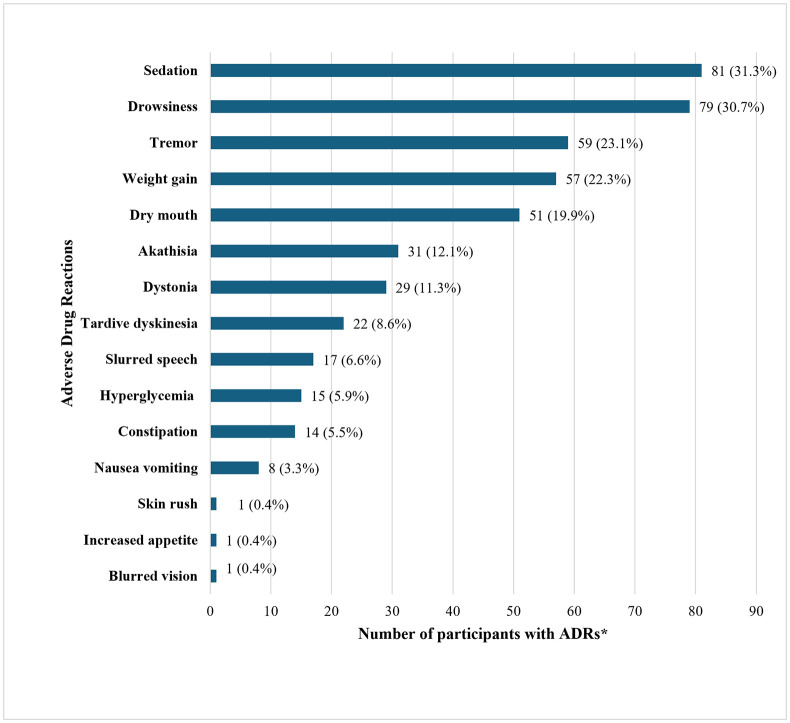
Commonly reported ADRs among patients taking antipsychotics at Psychiatric Clinic of MRRH. *The ADRs are not mutually exclusive.

Sedation was the most frequently reported ADR (80 cases; 31.3%), followed by drowsiness (79; 30.7%), tremors (59; 23.1%), weight gain (57; 22.3%), and dry mouth (51; 19.9%). One participant experienced over 9 ADRs (Fig 2).

Based on the Naranjo ADR causality assessment scale, out of the 256 ADRs identified, the majority (174, 68.0%) were rated as probable whereas only 72 (28.1%) and 10(3.9%) were classified as possible and definite respectively.

### Severity and preventability of ADRs

The severity of the ADRs was evaluated using the Modified Hartwig and Siegel scale, which showed that 163 (63.7%%) of the ADRs were of moderate, and 93 (36.3%) were mild. None of the ADRs were rated as severe. Preventability of the ADRs was assessed using the Schumock and Thornton scale, revealing that 130 (50.8%) of the ADRs were definitely preventable, while 89(34.8%) where probabily preventable and 37(14.4%) were deemed nonpreventable.

### Factors associated with ADRs

At bivariate analysis level, factors that were statistically significant (*p* < 0.05) included the separated category under marital status and the combined use of both oral and IM routes of administration. Variables with p-value less than 0.25 at bivariate analysis were age, gender, marital status, employment status, type of disorder, duration with disorder (years), and route of administration.

In multivariate logistic regression analysis, only separated category of marital status (AOR = 2.01, 95% CI: 1.08–3.77; *p* value = 0.029), having bipolar disorder (AOR = 2.09, 95% CI: 1.07–4.07; *p* value = 0.031), and the concomitant use of both IM and oral antipsychotics (AOR = 1.95, 95% CI: 1.03–3.70; *p* value = 0.041) remained significantly associated with experiencing at least one ADR (Table 4).

**Table 4:** Logistic regression of the factors associated with ADR among patients taking antipsychotics at MRRH.

Variable	Category	ADR, n (%)		Bivariate analysis		Multivariate analysis	
		No	Yes	Crude OR(95% CI)	*p*-value	Adjusted OR (95% CI)	*p*-value
**Age (years)**	26-60	109 (36.0%)	194 (64.0%)	1			
	18-25	38 (46.9%)	43 (53.1%)	0.64 (0.39-1.04)	0.073		
	>60	12 (38.7%)	19 (61.3%)	0.89 (0.42-1.90)	0.763		
**Gender**	Female	81 (35.1%)	150 (64.9%)	1		1	
	Male	78 (42.4%)	106 (57.6%)	0.73 (0.49-1.09)	0.128	0.78 (0.50- 1.22)	0.277
**Level of education**	No formal education	12 (40.0%)	18 (60.0%)	1			
	Primary	49 (37.7%)	81 (62.3%)	1.10 (0.49-2.48)	0.815		
	Secondary	60 (42.3%)	82 (57.7%)	0.91 (0.41-2.03)	0.820		
	Tertiary	38 (33.6%)	75 (66.4%)	1.31 (0.57-3.01)	0.516		
**Marital status**	Single	69 (42.1%)	95 (57.9%)	1		1	
	Married/Cohabiting	69 (39.6%)	105 (60.4%)	1.10 (0.72-1.71)	0.651	1.13 (0.72- 1.77)	0.587
	Separated	21 (27.3%)	56 (72.7%)	2.58 (1.23-5.38)	0.012	2.01 (1.08-3.77)	**0.029**
**Employment status**	Formally employed	9 (26.5%)	25 (73.5%)	1			
	Self-employed	26 (43.3%)	34 (56.7%)	0.48 (0.19-1.21)	0.121		
	Unemployed	124 (38.6%)	197 (61.4%)	0.57 (0.26-1.26)	0.164		
**Estimated monthly income (in UGX)**	<300000	17 (36.2%)	30 (63.8%)	1			
	300000-900000	12 (41.4%)	17 (58.6%)	0.80 (0.31-2.07)	0.650		
	>900000	6 (33.3%)	12 (66.7%)	1.13 (0.36-3.57)	0.831		
**Total perceived social support score**	High	125 (38.1%)	203 (61.9%)	1			
	Medium	31 (37.8%)	51 (62.2%)	1.01 (0.62-1.67)	0.959		
	Low	3 (60.0%)	2 (40.0%)	0.41 (0.06-2.49)	0.333		
**Type of disorder**	Depression	23 (47.9%)	25 (52.1%)	1		1	
	Bipolar	64 (34.2%)	123 (65.8%)	1.77 (0. 93- 3.36)	0.082	2.09 (1.07- 4.07)	**0.031**
	Schizophrenia	47 (37.3%)	79 (62.7%)	1.55 (0.79-3.03)	0.203	1.55 (0.77-3.11)	0.222
	Substance use disorder	25 (46.3%)	29 (53.7%)	1.07 (0.49-2.33)	0.870	1.57 (0.67-3.71)	0.302
**Comorbidities**	None	145 (39.2%)	225 (60.8%)	1			
	One	13 (30.2%)	30 (69.8%)	1.49 (0.75-2.95)	0.255		
	Two	1 (50.0%)	1 (50.0%)	0.64 (0.04-10.38)	0.757		
**Duration with disorder (years)**		2 (1, 4)	3 (1, 6)	1.03 (1.00-1.06)	0.071		
**Type of antipsychotic**	First	121 (37.7%)	200 (62.3%)	1			
	Second	33 (40.7%)	48 (59.3%)	0.88 (0.54-1.45)	0.614		
**Route of administration**	Orally	138 (40.2%)	205 (59.8%)	1		1	
	IM	5 (41.7%)	7 (58.3%)	0.51 (0.14-1.84)	0.302	0.90 (0.27- 3.05)	0.866
	Both oral and IM	16 (26.7%)	44 (73.3%)	1.85 (1.004-3.41)	0.048	1.95 (1.03- 3.70)	**0.041**
**Counseled on ADRs**	No	20 (41.7%)	28 (58.3%)	1			
	Yes	139 (37.9%)	228 (62.1%)	1.17 (0.64 - 2.16)	0.612		

## Discussion

This study aimed to determine the prevalence, severity, and associated factors of ADRs among patients taking antipsychotics. The findings showed that 256 (61.7%) of patients experienced at least one ADR. The concomitant use of both oral and intramuscular (IM) antipsychotics, a diagnosis of bipolar disorder, and being separated from a spouse were all associated with higher odds of experiencing antipsychotic-related ADR.

Our study reported an ADR prevalence of 61.7% among patients on antipsychotic therapy, which was higher than the 51.9% prevalence previously documented in a study conducted in India, a middle income country, among patients receiving antipsychotics [18]. The higher prevalence observed in the present study may be explained by differences in patient characteristics, prescribing patterns, and healthcare systems. Patients in our setting may have been more likely to receive higher antipsychotic doses, combination therapy, or concurrent oral and intramuscular formulations. Variations in pharmacovigilance practices, and the availability of routine monitoring and follow-up services may have contributed to the observed difference in prevalence. This is particularly important to explain the higher prevalence of ADRs among outpatients because of the limited pharmacovigilance services including less monitoring and follow-up compared to inpatients. By contrast, the ADR prevalence in our study was markedly lower than the 91.8% documented by Ejeta et al. among psychiatric patients prescribed psychotropic medications at Mizan Tepi University Teaching Hospital in Ethiopia [19]. It is worth noting that while Ejeta et al examined ADRs across a wider spectrum of psychotropic agents, encompassing medications that alter mood, perception, behavior or cognition, the present study specifically focused on antipsychotics, a subset of psychotropic drugs. Among all ADRs recorded in this study, sedation emerged as the most common, which is consistent with the pharmacological profile of FGAs, the class predominantly prescribed among our participants. A similar pattern of sedation as among the leading ADRs has been documented in a studies conducted in Ethiopia [19,39].

Of the 256 ADRs identified, the majority (68.0%) were classified as probable, while 72 (28.1%) were mild and 10 (3.9%) were categorized as definite according to the Naranjo ADR causality scale. These finding are consistent with previous studies reporting that most ADRs among patients receiving antipsychotics were rated as probable, including 74.3% in Oman [40], 55% in India [21]. This observation may be explained by the fact that many participants fulfilled key criteria for a “probable” classification under the Naranjo scale, such as the presence of prior conclusive reports of the reaction, onset of the ADR following administration of the suspected drug, and recurrence of the reaction upon drug re-administration.

These findings highlight the need for active and coordinated pharmacovigilance systems, routine ADR screening, and enhanced training of mental health professionals to improve early detection, documentation, and management of antipsychotic-related ADRs. Across Sub-Saharan Africa, where mental health services are often constrained by limited resources and specialist workforce shortages, integrating ADR monitoring into existing mental health programs could improve treatment safety, adherence, and clinical outcomes. Globally, the findings reinforce the importance of close monitoring and management of ADRs among patients receiving antipsychotics.

The majority of identified ADRs (163, 63.7%) were classified as moderate in severity, while 93 (36.3%) were mild. The predominance of moderate reactions in this study was comparable to previous findings from Oman, where 74.0% of reported ADRs were also rated as moderate [40]. One possible explanation is the high use of typical (first-generation) antipsychotics among participants. These agents are generally associated with higher burden of clinically significant adverse effects, which may necessitate therapeutic interventions such as dose adjustment, temporary withholding, discontinuation, switching of medication, or the administration of adjunctive treatment. This may be explained by their strong dopamine D2 receptor antagonism, which is associated with a greater risk of extrapyramidal symptoms (EPS) such as parkinsonism, akathisia, dystonia, and tardive dyskinesia.

This study further demonstrated that 130 (50.8%) of the ADRs were classified as definitely preventable, 89 (34.8)% were probably preventable, and 37 (14.4)% as non-preventable. The predominance of definitely preventable reactions underscores important gaps in prescribing practices, monitoring, follow-up care, and highlights the critical role of a multidisciplinary healthcare team in minimizing medication-related harm. These findings reinforce the need for targeted prevention strategies, particularly among patients identified as being at high risk of ADRs such as patients who separated from their spouses, individuals diagnosed with bipolar disorder, and those concurrently receiving both intramascular and oral antipsychotics. For such patients, careful drug selection, individualized dosing, routine monitoring, and structured follow-up are essential. Practical preventive approaches include timely recognition and management of early symptoms, optimizing adherence to treatment regimens, and providing comprehensive patient counseling. These findings highlight the need to strengthen rational prescribing practices, medication review systems, and routine monitoring of patients receiving antipsychotic therapy.

The proportion of preventable ADRs observed in this study is lower than 97% reported in a study in India, where the use of atypical antipsychotics and limited polypharmacy may have facilitated clearer attribution and prevention of ADRs [41]. However, our findings indicate a higher proportion of preventable ADRs compared to other Indian studies, which is higher compared to proportions ranging from 21.6% to 45.9% in studies from India [21,42]. Many ADRs associated with atypical agents, such as weight gain, metabolic disturbances, sedation, and hyperprolactinemia, tend to develop gradually and can often be anticipated, monitored, and mitigated through routine clinical assessment, dose adjustment, lifestyle interventions, and appropriate drug selection.

Identifying factors associated with ADRs among patients taking antipsychotics is essential for recognizing individuals at higher risk, enabling closer monitoring, timely intervention, and ultimately improving patient safety and quality of life [43]. In this study, patients who had separated from their spouses were twice as likely to experience ADRs. This may be attributed to inadequate social support, particularly from family, which can result in poor adherence to prescribed medication, worsening psychiatric symptoms, relapse of psychotic episodes, and the need for higher antipsychotic doses to manage the symptoms, thus increasing the risk of ADRs [44]. Additionally, poor adherence may lead to incorrect medication use, suboptimal self-monitoring, and underreporting of ADRs.

The study also found that patients diagnosed with bipolar disorder were twice as likely to develop ADRs following antipsychotic treatment. Similar findings have been reported among 441 children and adolescents in Turkey where those with bipolar disorders had a higher likelihood of experiencing ADRs when on antipsychotics [45]. This association may reflect the use of more aggressive antipsychotic dosing strategies, as bipolar patients often present with acute psychotic episodes that require higher antipsychotic doses.

Moreover, co-administration of antipsychotics via both oral and IM routes can result in additive pharmacologic effects, leading to higher plasma drug concentrations and increased risk of ADRs. IM antipsychotic formulations typically have a long half-life, leading to prolonged exposure of the drug in the blood and when co-administered with oral antipsychotics, the probability of getting ADRs increases. Insufficient follow-up and monitoring of these patients further amplify this risk [46]. Consistent with this mechanism, our study found that patients receiving both IM and oral antipsychotics had nearly double the odds of experiencing ADRs.

These findings highlight the need for risk-based approaches to antipsychotic prescribing and monitoring, with particular attention to patients who may lack adequate social support, those diagnosed with bipolar disorder, and those receiving multiple antipsychotic formulations. Mental health programs should incorporate routine assessment of social determinants of health, strengthen psychosocial support services, and enhance patient and caregiver education to improve medication adherence and early recognition of ADRs. Prioritizing high-risk patients for closer follow-up and pharmacovigilance could improve treatment outcomes and reduce preventable medication-related harm.

### Strengths and limitations of the study

This study targeted patients on antipsychotics, a population inherently at high risk for ADRs, thereby addressing a critical area in mental healthcare and psychopharmacotherapy. The relatively large sample size of 415 participants provided sufficient power to accurately estimate the prevalence of ADRs. Standardized tools were employed to assess causality, severity, and preventability, strengthening the reliability of the findings. However, the cross-sectional design limits the ability to establish causality between antipsychotic use and ADR occurrence. Future research should adopt longitudinal study designs to determine cause-effect relationships. Being a single-center study, the findings may have limited generalizability, although they offer valuable insights for future multi-centered studies. Another potential limitation of this study was that the sample size calculation was based on a prevalence estimate from an Indian study because comparable local data were unavailable at the time of study design. However, this is unlikely to have affected the validity of the study findings, as the final sample size remained adequate after accounting for the study design and the anticipated non-response rate. Additionally, the assessment of baseline weight and glycemic status relied on documented medical records and patient recall, which may have introduced recall bias and incomplete data for some participants. Laboratory testing for hyperglycemia was not universally available for all participants due to resource constraints, potentially leading to under-detection of metabolic ADRs. Future prospective studies should incorporate standardized baseline assessments and routine laboratory monitoring to address these limitations*.* While standardized operational definitions were applied to all 15 ADR types, we acknowledge that assessment of subjective ADRs (e.g., sedation, drowsiness, dry mouth) relies on patient self-report, which may have introduced reporting bias. However, clinical examination and investigator assessment were employed to corroborate patient reports where possible*.*

## Conclusions

The current study showed that over 6 in 10 participants taking antipsychotics experienced at least one ADR. More than three quarters were moderate in severity. Separation from spouses, diagnosed with bipolar disorder and co-administering both IM & oral antipsychotics were shown to be significantly associated with ADRs among patients taking antipsychotics. This implies the need to strengthen monitoring and preventive strategies, particularly among these high-risk patient groups. Based on our findings, we recommend that active monitoring for ADRs is done amongst healthcare teams at the psychiatry department at MRRH and ensure psychoeducation for patients taking antipsychotics. Integration of clinical pharmacists into the psychiatry team to review medication regimens, monitor ADRs and guide appropriate interventions would also help to address this. Routine use of ADR monitoring tools like the National Drug Authority ADR form to actively monitor and report will enhance pharmacovigilance of ADRs thereby promoting improved patient outcomes. Further studies should comprehensively explore all antipsychotic related ADRs using robust methodologies.

## Supporting information

S1 ADR_Dataset The minimum dataset of the study data. (XLSX)

## Abbreviations

ADR: Adverse Drug Reaction
AOR: Adjusted odds ratios
CI: Confidence interval
FGA: First Generation Antipsychotics
HIV/AIDS: Human Immunodeficiency Virus/Acquired Immunodeficiency Syndrome
IM: Intramuscular
MRRH: Mbarara Regional Referral Hospital
MUST: Mbarara University of Science and Technology
TGA: First Generation Antipsychotics
SGA: Second Generation Antipsychotics
SSA: Sub-Saharan Africa
WHO: World Health Organization
