# Correction: Prevalence, severity and predictors of Antipsychotic-Related Adverse Drug Reactions at the Psychiatry Clinic of a Regional Referral Hospital in Uganda

**DOI:** 10.1371/journal.pone.0358548

**Published:** 2026-09-15

**Authors:** John Martin Tumwebaza, Sarad Pawar Naik Bukke, Bayapa Reddy Narapureddy, Radiana Makuza Kabera, Joel Sebisaalu, Amina Abubakar, Patrick Muasya Kitheka, Godwin Nimusiima, Awad Osman Abdalla Mohamed, Moses Muwanguzi, Scholastic Ashaba, Tadele Mekuriya Yadesa

There are errors in the author affiliations. The correct affiliations are as follows:

John Martin Tumwebaza^1,2^, Sarad Pawar Naik Bukke^1,3^, Bayapa Reddy Narapureddy^4^, Radiana Makuza Kabera^2^, Joel Sebisaalu^2^, Amina Abubakar^2^, Patrick Muasya Kitheka^2^, Godwin Nimusiima^2^, Awad Osman Abdalla Mohamed^5^, Moses Muwanguzi^6^, Scholastic Ashaba^6^, Tadele Mekuriya Yadesa^7^

**1** Department of Pharmacy, Faculty of Health Sciences, Victoria University, Kampala, Uganda, **2** Department of Pharmacy, Faculty of Medicine, Mbarara University of Science and Technology, Mbarara, Uganda, **3** Department of Pharmaceutics and Pharmaceutical Technology, School of Pharmacy, Kampala International University, Ishaka, Uganda, **4** Department of Public Health, College of Applied Medical Sciences, King Khalid University, Abha, KSA, **5** Department of Anaesthesia and Operations, College of Applied Medical Sciences, King Khalid University, Abha, Saudi Arabia, **6** Department of Psychiatry, Faculty of Medicine, Mbarara University of Science and Technology, Mbarara, Uganda, **7** Department of Clinical Pharmacy and Pharmacy Practice, School of Pharmacy, Kampala International University, Ishaka, Uganda.
